# Time Course of the Involvement of the Right Anterior Superior Temporal Gyrus and the Right Fronto-Parietal Operculum in Emotional Prosody Perception

**DOI:** 10.1371/journal.pone.0002244

**Published:** 2008-05-21

**Authors:** Marjolijn Hoekert, Leonie Bais, René S. Kahn, André Aleman

**Affiliations:** 1 BCN Neuroimaging Center, University of Groningen, Groningen, The Netherlands; 2 Department of Psychiatry, University Medical Center Utrecht, Utrecht, The Netherlands; University of Southern California, United States of America

## Abstract

In verbal communication, not only the meaning of the words convey information, but also the tone of voice (prosody) conveys crucial information about the emotional state and intentions of others. In various studies right frontal and right temporal regions have been found to play a role in emotional prosody perception. Here, we used triple-pulse repetitive transcranial magnetic stimulation (rTMS) to shed light on the precise time course of involvement of the right anterior superior temporal gyrus and the right fronto-parietal operculum. We hypothesized that information would be processed in the right anterior superior temporal gyrus before being processed in the right fronto-parietal operculum. Right-handed healthy subjects performed an emotional prosody task. During listening to each sentence a triplet of TMS pulses was applied to one of the regions at one of six time points (400–1900 ms). Results showed a significant main effect of Time for right anterior superior temporal gyrus and right fronto-parietal operculum. The largest interference was observed half-way through the sentence. This effect was stronger for withdrawal emotions than for the approach emotion. A further experiment with the inclusion of an active control condition, TMS over the EEG site POz (midline parietal-occipital junction), revealed stronger effects at the fronto-parietal operculum and anterior superior temporal gyrus relative to the active control condition. No evidence was found for sequential processing of emotional prosodic information from right anterior superior temporal gyrus to the right fronto-parietal operculum, but the results revealed more parallel processing. Our results suggest that both right fronto-parietal operculum and right anterior superior temporal gyrus are critical for emotional prosody perception at a relatively late time period after sentence onset. This may reflect that emotional cues can still be ambiguous at the beginning of sentences, but become more apparent half-way through the sentence.

## Introduction

Besides the semantic meaning of words, features such as intonation, loudness and accents in speech may convey crucial information about the emotional state of the speaker. The term emotional prosody describes these non-linguistic cues in spoken language. The ability to perceive emotional prosody is crucial for understanding someone's true communicative intent and thereby for adequate social functioning [1]. Emotional prosody depends on intact functioning of the right hemisphere, although some studies suggest that it may also involve left hemisphere regions [2]–[7]. Imaging and patient studies have yielded discrepant data with respect to lateralization and location of brain regions contributing to the process of emotional prosody perception. Most of these studies show right (inferior or orbital) frontal [3], [6]–[13] and right temporal regions [5], [7]–[9], [11], [12], [14]–[21]. Others show bilateral frontal [2]–[7], [21], left temporal [3], [8] and right inferior parietal lobule [4], [13] involvement.

To further delineate the network of emotional prosody recognition, in the present experiment both a frontal and temporal location were targeted with transcranial magnetic stimulation (TMS). In TMS, brief magnetic pulses are delivered that penetrate the skull and disrupt neural processing in a non-invasive, reversible way [22]. Exact TMS sites for the present study were based on imaging studies that used sentences as stimuli and on a recent TMS study [23]. In the latter study increased reaction times were observed after modulation of the right fronto-parietal operculum [23]. This was a specific effect, only observed for a condition requiring emotional prosody discrimination, and not for a condition that required discriminating emotional semantics. Involvement of the right fronto-parietal operculum in the perception of emotional prosody was also found in other studies [3], [5], [7], [21].

The right fronto-parietal operculum forms part of the secondary somatosensory cortex. It has been suggested that knowledge about emotions expressed by other people might rely on a situation of body states associated with that emotion in the perceiver [24]. According to this theory, explicit recognition of an affective state in others depends on recruitment of one's own somatic expression representation associated with such emotion [24]. Activation of the somatosensory representation allows the observer to simulate the perceived emotion and evaluate and ‘feel’ it. The involvement of right somatosensory cortices in emotion recognition has been argued to occur irrespective of modality and of specific emotion [25]. Recent evidence from TMS studies shows that right somatosensoric areas, like the fronto-parietal operculum, may be specifically involved in evaluating withdrawal emotions [23], [26]. Not only the right fronto-parietal operculum but also the right anterior superior temporal gyrus seems to play a role in the perception of emotional prosody [3], [12], [21]. The anterior part of the superior temporal gyrus has been found to be a voice-selective area [27]. Regions along the anterior superior temporal gyrus show greater response to human voices as compared to non-vocal sounds [27]. This region has also been shown to be involved in both the production and perception of singing [28].

Although a large number of studies have tried to elucidate the brain regions involved in emotional prosody perception (resulting in discrepant data), only a few studies have looked at the time pattern of emotional prosody processing. To help in reconciling the controversial data from previous patient and imaging studies, we focused on both right fronto-parietal operculum and right anterior superior temporal gyrus in order to investigate their potentially different roles in the process of emotional prosody perception. Indeed, both right fronto-parietal operculum and right anterior superior temporal gyrus might be critical for the processing of emotional prosody, albeit possibly not for the same functional reasons. The multi-step process of emotional prosody perception has been described by Schirmer and colleagues, comprising the following mental processes in temporal order: 1) analyzing the acoustic elements of vocalizations, 2) deriving emotional significance from a set of acoustic cues, 3) applying emotional significance to higher order cognition [29]. A fourth component could be the integration of emotional prosody in language processing.

Sack and colleagues introduced a procedure to map time points at which different brain regions are critical for information processing [30]. This procedure allowed us to shed light on the precise time course of the contribution of the right anterior superior temporal gyrus and the right fronto-parietal operculum in emotional prosody perception. We expected to find the following time course of involvement of the two areas that were focused on in the present study. The right anterior superior temporal gyrus has been assumed to have a prominent role in the extraction of slow acoustic elements [4], [19], [27], [29], [31]. This area will be involved at the beginning of the time-path in step 1 of the multi-step process described above. Later the perceived emotion is represented in the somatosensory right fronto-parietal operculum where simulation provides information about the specific emotion [3], [32], part of step 2.

Aim of the present study was to analyze the effect of triple-pulse TMS over the right anterior superior temporal gyrus and the right fronto-parietal operculum at different time intervals from stimulus onset on the perception of emotional prosody. An additional active control TMS condition was included to test whether the effect is specific to the targeted region.

## Results

Reaction times and percentages correct were analyzed by repeated measures ANOVA, in which the factors were Site of stimulation (anterior superior temporal gyrus (STG), fronto-parietal operculum (FPO), POz), Time (6 points, varying from 400 to 1900ms, with 300 ms in between) and Emotion (fear, sad, happy). Only reaction times for correct trials were included in the analyses. Due to a high percentage correct over all subjects (84%) the results are based on the majority of data collected. *Post hoc* analyses were performed using pair-wise comparisons. Effects were tested at a significance level of p<.05. At the beginning of each transcranial magnetic stimulation (TMS) session subjects were asked to complete the positive and negative affect schedule (PANAS) to evaluate their current affective state [33]. This was done to be able to correct for potentially different affective states that could influence the performance on an emotion task. A 3×2 repeated measures ANOVA with Site of stimulation (aSTG, FPO, POz) and scores on the PANAS scale (positive scale, negative scale) revealed no significant differences between the three TMS sessions, *F* (2,18 ) = 1.22, p = .32. This means that we can assume that there was no significant effect of mood on the reaction times [33]. Differences in reaction times during TMS conditions were not attributable to differences in mood state between the TMS conditions. Gender was added as a between subjects factor in the repeated measures ANOVA, but no significant differences were found across gender groups, *F* (1, 8) = .47, p = .51.

### Reaction times during triple-pulse rTMS at the fronto-parietal operculum and the anterior superior temporal gyrus

A 2×6×3 repeated measures ANOVA with Site of stimulation (aSTG, FPO), Time (6 points, from 400 to 1900ms) and Emotion (fear, sad, happy) as independent variables, showed that there was no significant difference in reaction times between the two sites of stimulation, *F* (1,13) = .79, p = .39. There was a main effect of Time, *F* (5, 65) = 31.3. p<.001. In addition, the quadratic term was highly significant, *F* (1, 13) = 69.4, p<.001. The quadratic trend reflects a peak in reaction times at a certain time point. Post hoc analysis revealed most strong interference of the triplet of TMS pulses at 1300 ms from onset of the stimulus. At this onset time the mean reaction time was significantly larger than at all other time points, mean differences varied from 114 ms to 346 ms, (p<.05).

**Figure 1:**
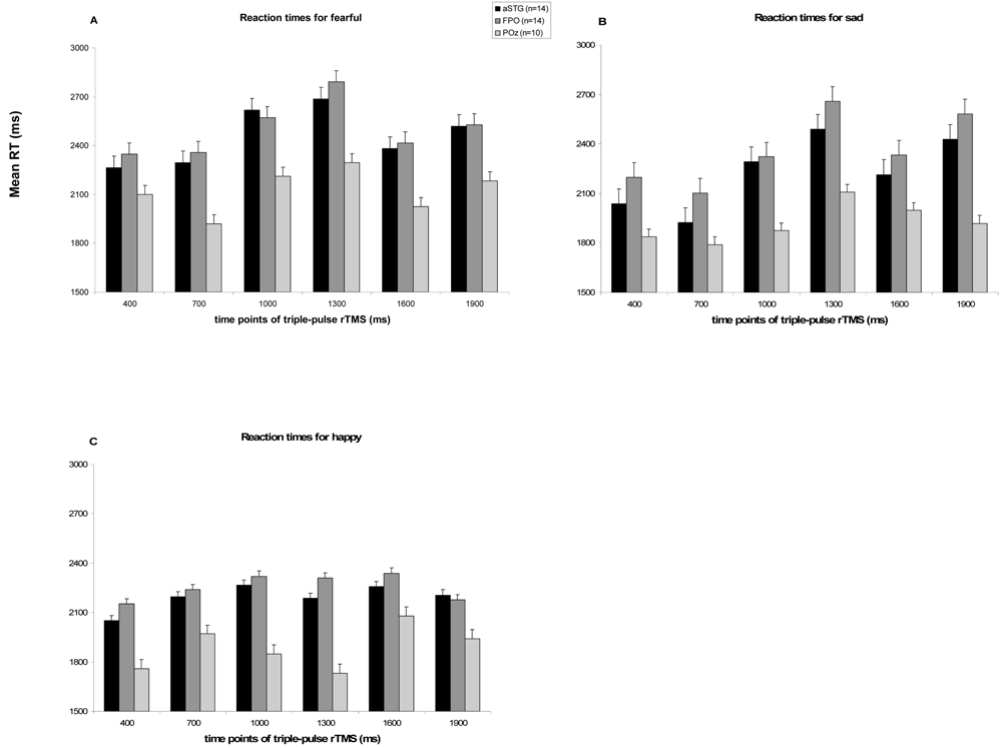
Reaction times on emotional prosody task during triple-pulse rTMS at different time-point at the right anterior superior temporal gyrus (aSTG, n = 14 subjects), the fronto-parietal operculum (FPO, n = 14 subjects) and at the POz-EEG location (n = 10 subjects) for detection of emotional intonations. A: reaction times for perception of fearful intonations, B: reaction times for perception of sad intonations and C: reaction times for detection of happy intonations.

The analysis also revealed a main effect of Emotion *F* (2, 26) = 7.3, p<.010. Longest reaction times were found for the detection of the emotion fear. Contrasts revealed that the difference between reaction times for correctly classifying ‘fear’ sentences compared to ‘happy’ sentences was significant *F* (1,13) = 26.04, (p<.05). Subjects were faster in the detection of happiness from intonations than in the detection of fear. The difference between performance on ‘fear’ sentences and ‘sad’ was also significant *F* (1,13) = 5.20, (p <.05). Subjects responded faster on ‘sad’ sentences than on ‘fear’ sentences. Furthermore there was a significant interaction between Emotion and Time *F* (10,130) = 6.1, p<.001. This indicates that the time of delivering the triplet of TMS pulses had a different effect depending on which emotion was expressed. To explain this interaction, reaction times per emotion over the 6 time-points were analyzed. Time of stimulation significantly affected reaction times for the Emotions ‘fear’ and ‘sad’, *F* (5, 65) = 12.7, p<.001 and *F* ( 5, 65) = 28.4, p<.001 respectively. For the Emotion ‘happy’ Time of stimulation was also significant but with a larger p-value, *F* (5, 65) = 2.6, p<.05. Whereas the strongest interference for ‘fear’ and ‘sad’ stimuli was observed at 1300 ms, this was not apparent for happy (see figure 1).

### Reaction times during triple pulse rTMS with the inclusion of active control condition POz

To test whether the results described in the above section are a real TMS effect, an additional active control condition over the occipital cortex (POz electrode location) was included. We can assume that this brain area is not related to emotional prosody. Inclusion of condition POz (10 participants), revealed a main effect of Site of stimulation *F*( 2,18) = 4.7, p<.05. Contrasts revealed that both right anterior superior temporal gyrus and right fronto-parietal operculum differed significantly from POz, *F* (1, 9) = 6.5, p<.05 and *F* (1, 9) = 8.9, p<.05 respectively. There was also a main effect of Time *F* (5, 45) = 21.2, p<.001 and a main effect of Emotion *F* (2, 18) = 8.6, p<.01 as was found in the anterior superior temporal gyrus versus fronto-parietal operculum analysis. Analyzing the data for separate emotions, revealed a significant effect of Site of stimulation for ‘fear’ *F* (2, 18) = 4.2, p<.05, and for ‘happiness’ *F* (2, 18) = 6.5, p<.01, but not for ‘sadness’ *F* (2, 18) = 2.6, p = 0.10. As can be seen in Table 1, reaction times were shorter for all three emotions in the POz condition, as compared to anterior superior temporal gyrus and fronto-parietal operculum. The interaction between Site of stimulation and Time was significant, *F* (10, 90) = 1.9, p = .05, with stronger effects of Time in the anterior superior temporal gyrus and fronto-parietal operculum conditions than in the POz condition (see figure 1). There was no interaction between Site of stimulation and Emotion.

**Table 1:** Reaction times during triple-pulse rTMS for the three conditions

	aSTG (*n* = 14)			FPO (*n* = 14)			POz (*n* = 10)		
	fearful	sad	happy	fearful	sad	happy	fearful	sad	happy
400 ms	2264 (461)	2037 (570)	2051 (391)	2347 (537)	2198 (549)	2152 (364)	2100 (444)	1837 (561)	1760 (430)
700 ms	2295 (449)	1923 (519)	2195 (412)	2358 (615)	2102 (497)	2238 (461)	1919 (514)	1790 (516)	1970 (566)
1000 ms	2618 (537)	2293 (669)	2268 (506)	2571 (648)	2323 (655)	2320 (421)	2212 (643)	1875 (605)	1848 (434)
1300 ms	2687 (398)	2490 (741)	2186 (399)	2793 (636)	2660 (732)	2309 (467)	2295 (623)	2107 (695)	1732 (421)
1600 ms	2381 (497)	2215 (527)	2256 (432)	2415 (530)	2334 (519)	2339 (556)	2024 (513)	1998 (601)	2080 (566)
1900 ms	2518 (552)	2428 (592)	2207 (532)	2528 (595)	2583 (681)	2176 (454)	2182 (568)	1919 (634)	1940 (617)

No significant differences were observed between fronto-parietal operculum (FPO) and anterior superior temporal gyrus (aSTG) with regard to reaction times. Reaction times on POz were significantly different from both FPO and aSTG. Reaction times after triple-pulse rTMS at 1300ms after onset of the sentence were significantly higher than at all other time-points.

With regard to accuracy of responses, inclusion of the active control condition did not reveal significant effects of Site of stimulation, indicating that on accuracy measures no differences were found between the Sites. A significant interaction between Emotion and Time was found, paralleling the results for the reaction times analysis, *F* (10,90) = 5.7, p<.001. This reflects that the time of delivering the triplet of pulses had a different effect depending on which emotion was expressed. Data are presented in table 2.

**Table 2:** Emotion identified correctly (%) during triple-pulse rTMS for the three conditions

	aSTG (*n* = 14)			FPO (*n* = 14)			POz (*n* = 10)		
	fearful	sad	happy	fearful	sad	happy	fearful	sad	happy
400 ms	75.1 (24.9)	96.9 (7.2)	95.4 (9.4)	78.0 (14.1)	89.0 (9.9)	96.1 (6.3)	76.6 (21.6)	93.9 (8.7)	97.6 (5.1)
700 ms	64.5 (15.3)	90.4 (12.9)	84.7 (12.4)	70.9 (13.6)	92.1 (9.2)	86.6 (11.4)	69.5 (18.8)	86.4 (13.7)	95.2 (6.2)
1000 ms	60.1 (14.8)	92.1 (10.5)	89.4 (8.3)	62.7 (15.4)	93.0 (9.4)	90.4 (10.0)	66.5 (11.9)	91.4 (10.2)	90.2 (9.8)
1300 ms	69.4 (15.4)	92.9 (8.0)	85.6 (10.1)	65.7 (22.3)	96.6 (5.6)	86.7 (11.4)	71.5 (18.6)	95.2 (6.2)	85.0 (12.8)
1600 ms	75.2 (25.3)	87.9 (6.9)	84.9 (14.8)	81.4 (18.8)	84.1 (13.3)	82.9 (17.5)	85.3 (14.1)	81.4 (12.1)	83.6 (14.7)
1900 ms	75.2 (21.8)	94.7 (9.4)	96.6 (5.6)	72.4 (18.3)	99.1 (3.2)	97.3 (5.4)	74.9 (22.8)	97.6 (5.1)	95.2 (6.2)

No significant differences were found on percentages correct between the three sites, fronto-parietal operculum (FPO), anterior superior temporal gyrus (aSTG) and POz (EEG electrode site).

## Discussion

To our knowledge this is the first study investigating the time course of processing emotional prosody in natural sentences. This study demonstrates that triple-pulse transcranial magnetic stimulation (TMS) over the right anterior superior temporal gyrus as well as the right fronto-parietal operculum interferes at critical time points with the process of emotional prosody recognition from spoken sentences. This effect was significantly stronger after TMS over the fronto-parietal operculum and the anterior superior temporal gyrus than in the active control condition, stimulation over the occipital cortex. The most critical time point was at 1300 ms after sentence onset. This effect was apparent for withdrawal, but not for the approach emotion. The strong delay in response times at 1300 ms was found for detection of vocally expressed ‘fear’ and ‘sadness’, but not for ‘happiness’. We did not find a difference between right anterior superior temporal gyrus and right fronto-parietal operculum. Results showed a similar time pattern for both brain areas.

Our results are consistent with those of a recent study that targeted a right somatosensory area close to the fronto-parietal operculum but a bit higher, using TMS to investigate the perception of facial emotion expression [26]. Interestingly, this study found that TMS over that area interfered with the recognition of fear (a withdrawal emotion), but not with the recognition of happy (approach emotion) facial expressions [26]. Our findings are also a replication and extension of the study by Van Rijn and colleagues, who targeted the right fronto-parietal operculum with TMS and measured the effect on emotional prosody perception. They also found an effect on the recognition of withdrawal emotions (fear and sadness) but the performance on approach emotions (happiness and anger) was unaffected [23]. We have replicated the findings of these TMS studies on the effects of TMS at the right fronto-parietal operculum for detection of withdrawal emotions in emotional prosody [23]. In addition, however, we investigated the time course of the TMS interference.

Given previous evidence for the time course of emotional prosody recognition, 1300 ms after sentence onset seems rather late. However, the earlier studies used single words and syllables as stimuli. Results from an event-related potential study revealed that P200 is an event-related potential correlate of vocal emotional processing. Vocal emotions elicited a P200 smaller than neutral expressions, representing early differentiation of basic vocal emotional expressions and neutral vocal expressions [34]. In another event-related potential study that tested pre-attentive, vocal emotion processing using an oddball paradigm, emotionally (angry and happy) and neutrally spoken syllables ‘dada’ were presented as standards and deviants [35]. The difference in mismatch negativity amplitude between emotional and neutral vocalizations peaked around 350 ms, suggesting that at this time point the primary acoustic information and derived emotional significance had been integrated. In an event-related potential study using words, results suggested that emotional prosody is processed around 360 ms [36]. In this study, the duration of the stimuli was around 761 ms on average, and there were only two emotional categories spoken by one female actress. It is expected that under natural circumstances, as in the present study, the time window is prolonged. We used complete sentences with a mean duration of 2042 ms, three emotional categories and spoken by multiple male and female actors. It is noteworthy that in both studies, the peak of the process takes place halfway through the stimuli.

Our results did not reveal different time patterns of involvement of both brain areas, i.e., right anterior superior temporal gyrus and right fronto-parietal operculum. One possible explanation is that the processes ‘extraction of slow acoustic elements’ and ‘simulation of the perceived emotion’, ascribed to the right anterior superior temporal gyrus and the right fronto-parietal operculum respectively, take place at the same time. These processes then interact with the putatively more frontal process of categorization. Stimulation of one site would result in widespread activation along the functional network and thus in activation of the other site as well. ‘Recurrent neural networks’ have been found for visual information processing [37] and consist of a feed-forward-system, e.g. anterior superior temporal gyrus to fronto-parietal operculum, and a feedback system, e.g. fronto-parietal operculum to anterior superior temporal gyrus. Auditory information can be exchanged very quickly between the anterior superior temporal gyrus and the fronto-parietal operculum. In this way both areas can be crucially involved in the process of emotional prosody perception at the same time.

A functional network of brain areas involved in phonological processes has been described in a recent meta-analysis on left hemisphere language areas [38]. In this study an area that corresponds to the fronto-parietal operculum, the rolandic operculum, has been described as part of the audio-motor loop. In this audio-motor loop the rolandic area is in charge of a motor representation of auditory stimuli to guide comprehension [38]. The fronto-parietal operculum forms part of the somatosensory cortex comprising representations of the lips, jaw and tongue. In emotional prosody perception these representations may be evoked to simulate the perceived auditory emotion. This simulation guides the comprehension of the acoustic properties of the human voice, that are processed in the anterior superior temporal gyrus at the same time. A recent imaging study provides evidence that the fronto-parietal operculum acts in an audio-motor loop to guide comprehension of emotional prosody. This study demonstrated that passive listening to nonverbal emotional vocalizations activates a network of premotor cortical regions involved in the control of facial movement [39]. This is consistent with Damasio's conceptualization of an “as-if body loop”, i.e. a neural internal simulation that uses the brain's body maps, but bypasses the actual body [40].

Another explanation could be inadvertent stimulation of the non-targeted area due to proximity of the right anterior superior temporal gyrus and the right fronto-parietal operculum. The mean distance between the two sites of interest was however 28 mm with a small SD of 4.9 mm, which is large enough to assume that stimulating one area does not directly affect the other [41].

Finally, it should be taken into account that the clicking sound of the TMS coil may have distracted the subject from the task and that this lowered performance. We cannot rule out this possibility, but there is enough reason to believe that there was (also) a real TMS effect. The results of our study provide evidence that the clicking alone cannot completely explain the delays in reaction times. Although the anterior superior temporal gyrus is closer to the ear, reaction times to stimulation of the right fronto-parietal operculum were even slower than with stimulation to the anterior superior temporal gyrus. A possible effect of the clicking sound can not explain this sufficiently. Moreover, the time pattern was not found for all emotions, if slower performances would be a result of acoustic interference of the clicking sound, we may have expected generalized effects. Van Rijn and colleagues also stimulated the right fronto-parietal operculum and observed a difference in emotional prosody recognition between sham stimulation (no magnetic stimulation but with the characteristic ‘click’) and real stimulation [23]. This suggests that the TMS-effect is distinct from a mere effect of the click-sound. The inclusion of the active control condition also suggests that the effect we found should be attributed to the direct magnetic stimulation of the brain. Reaction times in the active control condition were significantly shorter than in two areas that have been related to emotional prosody processing. Stimulation of right anterior superior temporal gyrus and right fronto-parietal operculum resulted in longer reaction times than stimulation of an area that has not been associated with emotional prosody.

The present study confirms that both the right anterior superior temporal gyrus and the right fronto-parietal operculum are involved in the process of emotional prosody perception. Moreover, the results reveal a relatively late critical time of involvement of both regions for recognizing emotional prosody in entire sentences. This may reflect that emotional cues can still be ambiguous at the beginning of sentences but become more apparent half-way through the sentence. Our study forms the first step in disentangling the temporal involvement of different brain areas in the process of emotional prosody recognition in natural speech.

## Materials and Methods

### Subjects

Fourteen healthy subjects (aged 19 to 41, 7 females) participated in the first two conditions (right anterior superior temporal gyrus and right fronto-parietal operculum). Ten (aged 19 to 29, 6 females) of these 14 also participated in the third active control condition (midline parietal-occipital junction EEG site, POz). All were right-handed, measured with the Edinburgh Handedness Inventory and had an adequate understanding of Dutch (comprehension and reading). Participants were checked for TMS and MRI exclusion criteria. They were given extensive written and oral explanation of the procedures and signed an informed consent. The experiment was conducted in accordance to the Declaration of Helsinki and with local ethics committee approval (University Medical Centre Groningen).

### Experimental setup

**Figure 2:**
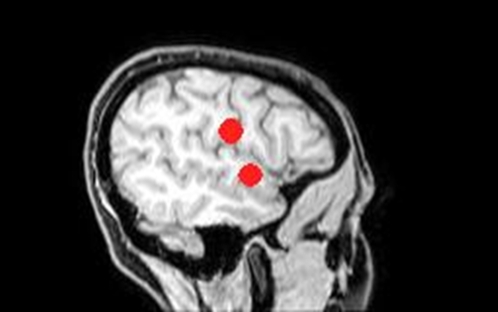
This figure shows the two TMS locations that are targeted in the present study drawn in MRIcro, the fronto-parietal operculum and the anterior superior temporal gyrus.

A neural navigator (NeNa) was used to locate the intended stimulation areas of each participant [42]. This frameless stereotaxy device uses a structural MRI scan of a person's head to guide a TMS coil to the proper region on the skull. Anatomical MRI scans were made on a Philips Intera 3 Tesla MR-system at the BCN-Neuroimaging Center, Groningen. Regions of interest were drawn in MRIcro a few days before the TMS experiment by the primary investigator and checked by another researcher until the two reached consensus (see figure 2). Number of voxels per region of interest was ca. 120, 1 to 2 cm^2^, in accordance with the size of the region TMS affects [43]. The fronto-parietal operculum was defined as the inferior peri-central sulcus area, at the border of the frontal and parietal lobes, marked by the central sulcus, situated at the bottom of the central sulcus (covering both precentral as well as postcentral regions) above the lateral sulcus, in accordance with the study of van Rijn [3], [23]. Talairach coordinates were ca. (53, −6, 14) [44]. The right anterior superior temporal gyrus was defined as approximately 1.5 cm posterior to the temporal pole, under the precentral sulcus, bordered by the lateral sulcus and superior temporal sulcus, based on imaging studies [5], [19], Talairach coordinates ca. (52, −4, −4) [44]. The additional TMS condition, EEG location POz could be marked on an EEG cap using the international 10–20 system, therefore no anatomical determination was needed. Talairach coordinates for this electrode location were approximately (0, −95, 16) [44]. At the beginning of the TMS sessions the right fronto-parietal operculum and the right anterior superior temporal gyrus were targeted and marked on an EEG-cap [45]. Mean distance between right fronto-parietal operculum and right anterior superior temporal gyrus measured on the EEG cap was 28 mm (SD 4.9 mm).

Triple-pulse TMS (tpTMS) was applied using a Medtronic MagPro X100 stimulator (Medtronic, Skovlunde, Denmark) with a figure-of-eight coil (diameter 75 mm), with a dynamic cooling device (Cool-B65). The coil was maintained in a constant position with the Medronic coil arm. The orientation of the coil was set with the handle pointing downwards, making an angle of 90° with the midline of the head.

### TMS Procedure

At the beginning of each TMS session, subjects completed the positive and negative affect schedule (PANAS), to assess their current affective state [33]. This self-assessment questionnaire consists of 10 positive (interested, excited, strong, enthusiastic, etc.) and 10 negative affects (distressed, upset, guilty, etc). All items have to be rated on a scale from 1 to 5, based on the strength of emotion where 1 = “ very slightly or not at all,” and 5 = “extremely”. Participants were seated in a comfortable chair, in front of a computer screen and fitted with an EEG cap to allow marking of stimulation sites. First, individual motor thresholds (MT) of the (preferable) left hemisphere were determined using the thumb (abductor pollicis brevis) movement procedure [46]. Stimulation intensity was set at 90% MT (mean 45.6 (SD 6.9)). First, participants completed a training session of 6 sentences. The entire procedure was completed in three sessions on separate days, around the same hour. Order of the experimental blocks and the two brain areas of interest (right fronto-parietal operculum and right anterior superior temporal gyrus) were counterbalanced over subjects. The extra active control condition was included later (in 10 subjects), this area was always stimulated in the last session.

A triplet of TMS pulses was given at various onset times per stimulus to identify the time points at which right anterior superior temporal gyrus and right fronto-parietal operculum are critical for processing emotional prosody. In every tpTMS run, the TMS coil was placed over the area of interest while the prosodic discrimination of emotion task was completed. During each trial, a triplet of three single TMS pulses was applied with an inter-pulse-interval of 100 ms, based on a recent TMS study [30]. In an event-related potential study, a peak was found around 360 ms, under voluntary processing of emotional prosody in words [36]. In a pilot study mean reaction time for detection of emotions was 2389 ms (SD 884) without TMS. Duration of response preparation is ±400 ms. Based on these data, the onset times of tpTMS in the present study varied between 400 and 1900 ms, in steps of 300 ms. There was a total of 8 experimental blocks of the same length during which tpTMS was applied at different pseudo-randomized time points on the scalp positions. The number of sentences per emotion per time-point was 8. Every ten seconds a new sentence was presented. Minimum time between triplets of pulses was 8.5 seconds, enough to avoid carry-over effects [22]. The procedure for the right fronto-parietal operculum and right anterior superior temporal gyrus TMS sessions took 90 minutes at maximum. The same design was used in an extra session, for the active control condition, i.e. real TMS was performed to this area but we did not expect effects on the task, because this area is not related to emotion in language. During this condition, stimulation was applied with the centre of the coil at the POz electrode site, the duration was 60 minutes. This is 30 minutes less than in the other two TMS conditions, because no neuronavigation was needed.

### Emotional prosody task

Sentences of neutral content pronounced in an emotional (anxious, sad or happy) tone of voice by male and female actors were used as stimuli. The task was developed and presented using Eprime software [47]. These were selected from three different validated prosody tests [48]–[50]. All sentences were Dutch, examples are, “The old car drives through the streets of the capital” and “Jan has been to the hairdresser”. The sentences were tested by ten separate subjects. The digitized stimuli were of approximately equal length and presented through two computer sound boxes (mean duration 2042 ms (SD 755)). During listening, the names of the emotions to be discriminated were presented on the computer screen. The visual presentation of the answer choices was included to aid subjects as to which categories they were to choose from. Without the visual presentation, subjects would have to memorize the different categories, and the task would have a stronger working memory component. Participants were instructed to use the index finger of their right hand to make a ‘fear’ response on a keypad, the middle finger to make a ‘sad’ response or the thumb for a ‘happy’ response as soon as they identified the emotion expressed in the tone of voice. Speed and accuracy were stressed as objectives.
